# Ventilation

**Published:** 1871-11

**Authors:** 


### VENTILATION.

Let a shaft run from the cellar through every room, and beyond the roof, having an aperture near the floor and near the ceiling, with an upward draft of warm air from a furnace or stove in the cellar. This arrangement seems to act well in one of the school buildings in Providence, Rhode Island. The practical solution of the subject of the ventilation of buildings has not as yet been satisfactorily reached; meanwhile there is one easy way of a good ventilation of the family apartment; it has several advantages, and is readily understood by all. Keep a rousing fire on the hearth, such as Dixon's Low Down Grate furnishes, and let the door of the apartment leading from the hall be kept open pretty much all the time. Two things will always follow: there will be no ill results from over-heated apartments, and the foul air of the room will be steadily carried up the chimney to the skies above; a little more fuel will be consumed, but there would be overbalancing advantages on the score of good health.

### FIRES IN THE FALL.

Comfort, health, and life are sometimes sacrificed by tidy housekeepers, in endeavoring to put off kindling fires in the autumn as long as possible, from the dread of ashes and coal dust flying about and settling on their beautiful furniture and handsome carpets, with the result that the family are kept in a shiver for hours, unless they can manage to hurry out into the sunshine as soon as breakfast is over. This uncomfortably cold condition of the body, lasting for an hour or more, is quite sufficient to bring on an attack of inflammation of the lungs in old or feeble persons; it is a risk that never should be taken. Only think of it — the risk of a dangerous and very often fatal ailment, involving an immense amount of solicitude in the family, as well as painful suffering of the patient, and doctors' bills beside, for the mere trouble of kindling a little fire, costing but a few cents !

There ought to be a thermometer in every family room, which all the members should be taught to consult habitually; and whenever it points to sixty-eight degrees, any time before steady fires are in operation, a brisk blazing fire should be kindled on the hearth, for the benefit of all who need its warmth; the very sight of such a fire on entering an apartment, of a cool autumn morning, carries with it a peculiar satisfaction, made up of a feeling of coziness and comfort. It is curious to note that in the early autumn we feel chilly on entering a room at sixty-eight; but in midwinter the same degree of warmth, on entering an apartment, is felt to be oppressive.

